# Supplementary material for: Single-Particle Tracking of Thermomyces lanuginosus Lipase Reveals How Mutations in the Lid Region Remodel Its Diffusion
Source: Biomolecules. 2023 Mar 31;13(4):631. doi: 10.3390/biom13040631 (PMC10135605; doi:10.3390/biom13040631)
Supplement: Supplementary file 1 [file biomolecules-13-00631-s001.zip › biomolecules-2244686-supplementary.pdf]

Supplementary Information

Single-Particle Tracking of *Thermomyces lanuginosus* lipase diffusion reveals how mutations in the lid region remodel its diffusion

Josephine F. Iversen[1,2,†], Søren S.-R. Bohr[1,2,†], Henrik D. Pinholt[1,2,3], Matias E. Moses[4], Lars Iversen[4], Sune M. Christensen[4], Nikos S. Hatzakis[1,2]*, Min Zhang[1,2]*

[1] Department of Chemistry & Nanoscience Center, University of Copenhagen, Thorvaldsensvej 40, 1871 Frederiksberg, Denmark

[2] Novo Nordisk Foundation Centre for Protein Research, Faculty of Health and Medical Sciences, University of Copenhagen, Blegdamsvej 3B, 2200 Copenhagen, Denmark

[3] Department of Physics, Massachusetts Institute of Technology, Cambridge, MA 02139, USA

[4] Novozymes A/S, 2800 Kgs. Lyngby, Denmark

[†] These authors contributed equally to this work.

*Correspondence: hatzakis@chem.ku.dk (N.S.H.); min.zhang@chem.ku.dk (M.Z.)

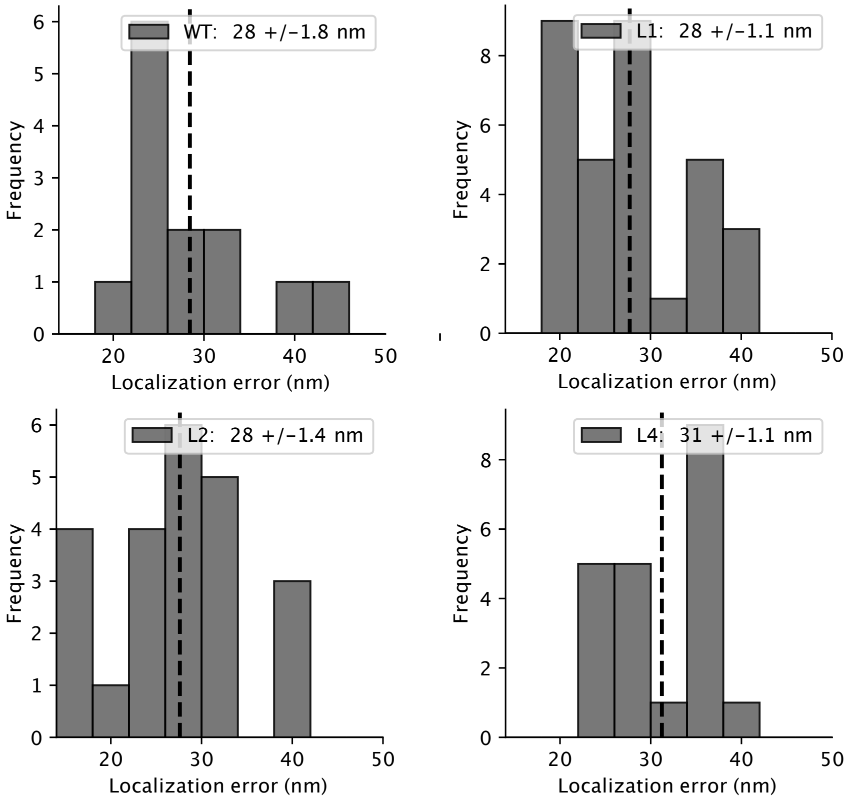

Figure S1. Localization errors of different types of TLLs.

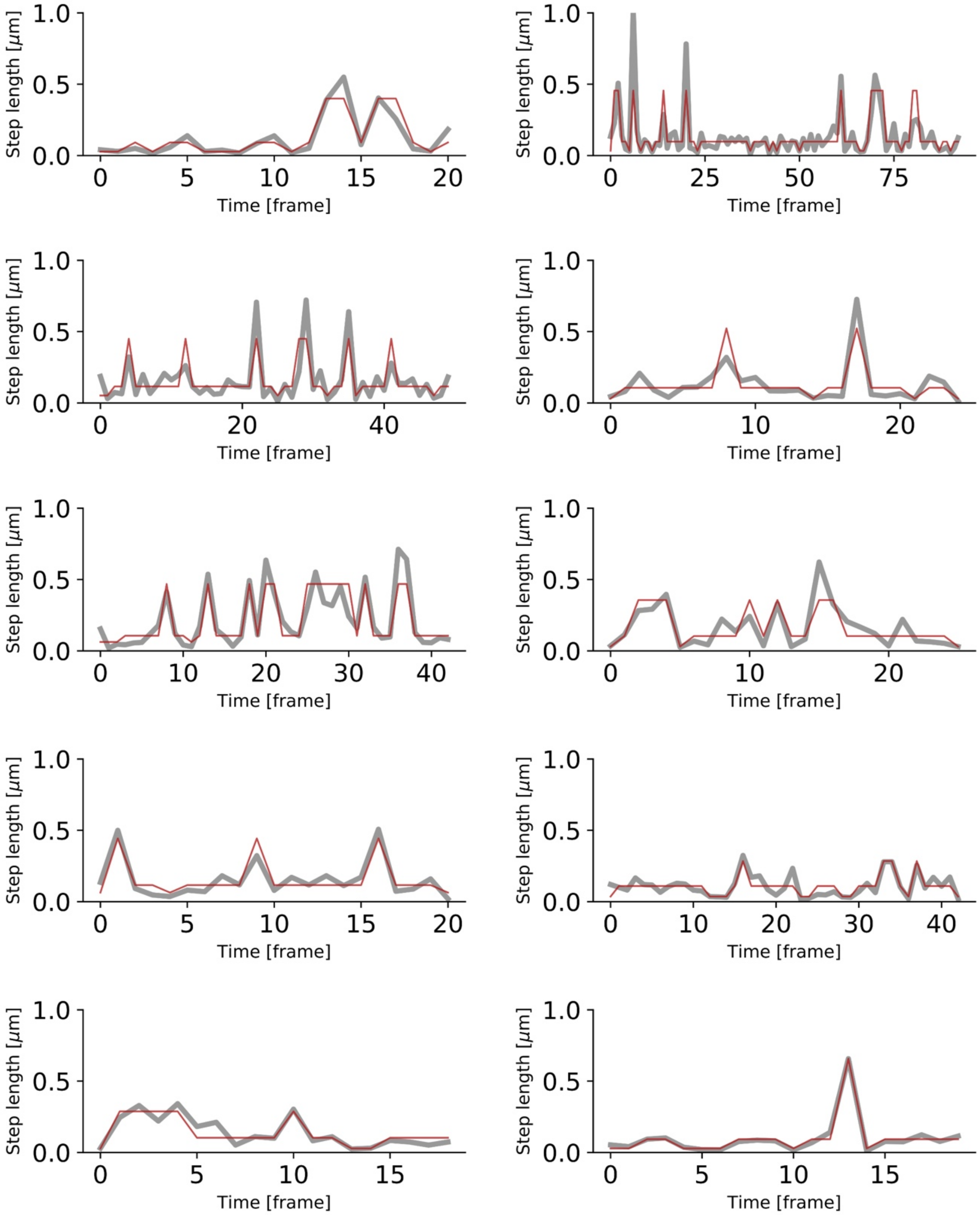

Figure S2. Representative traces (gray) and their HMM idealized states (red).

Table S1. Quantification of average diffusion with standard deviation for lipase variants in the application system.

Lipase	Total tracks	Average Diffusion Coefficient [μm²/s]
L1	20,793	0.0561 ± 0.0480
L2	8590	0.0524 ± 0.0416
L4	26,320	0.0764 ± 0.0420
WT	16,792	0.0518 ± 0.0319

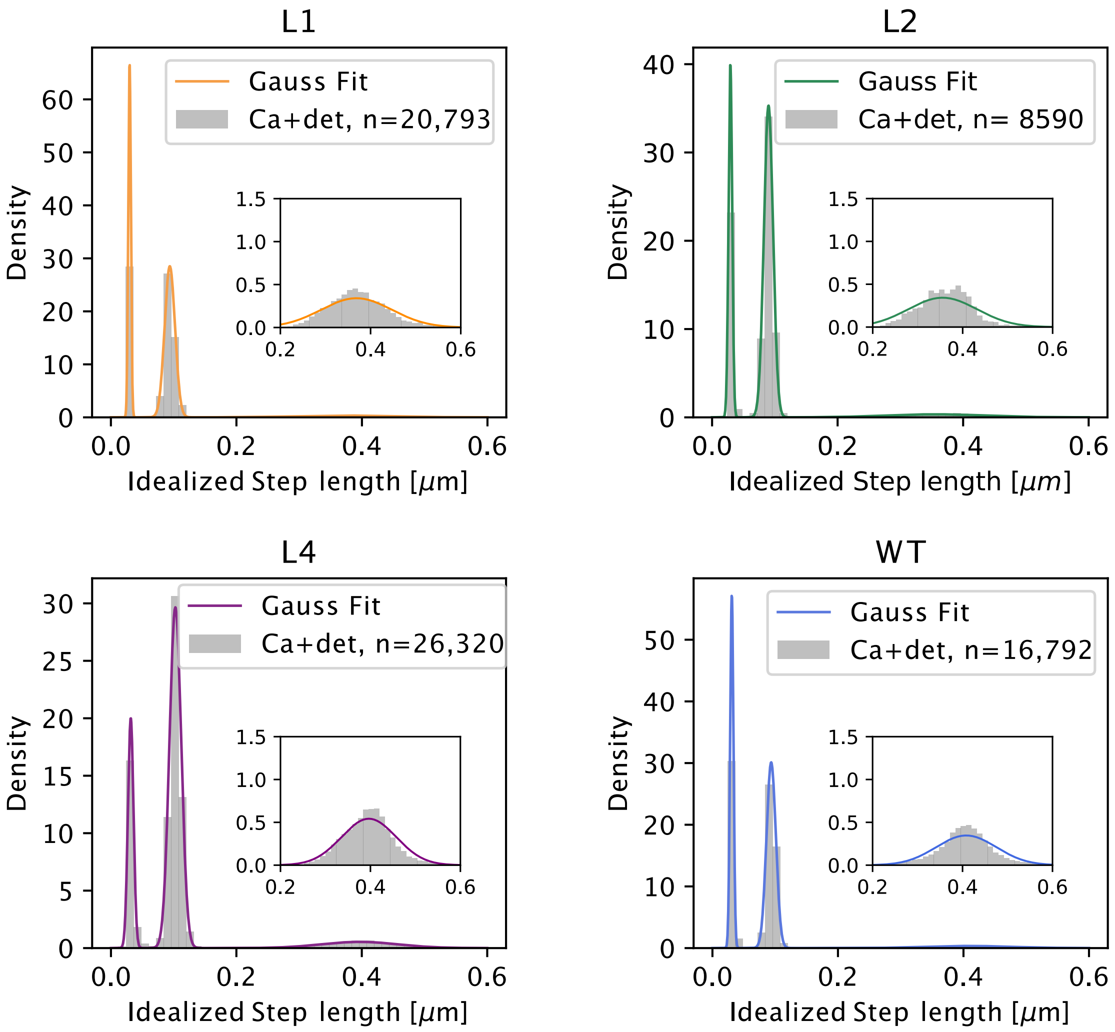

Figure S3. Gaussian mixture model fitted with three Gaussian functions to the idealized step length distribution after HMM segmentation for TLL variant in application condition. Inset: zoom in on the fast states.

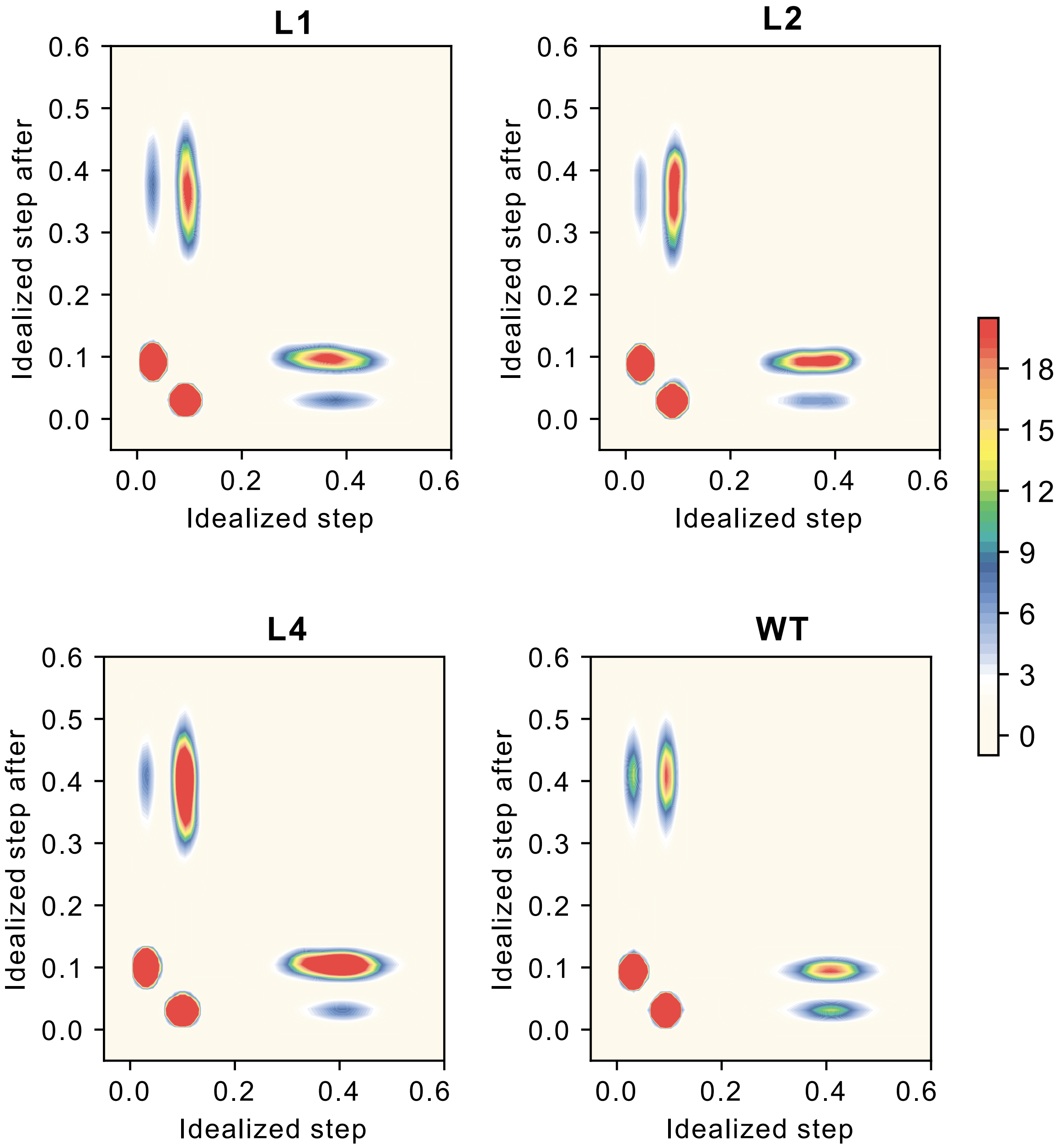

Figure S4. Transition density plots (TDPs) of TLL variants in the application condition. Color bar means the relative frequency (%).

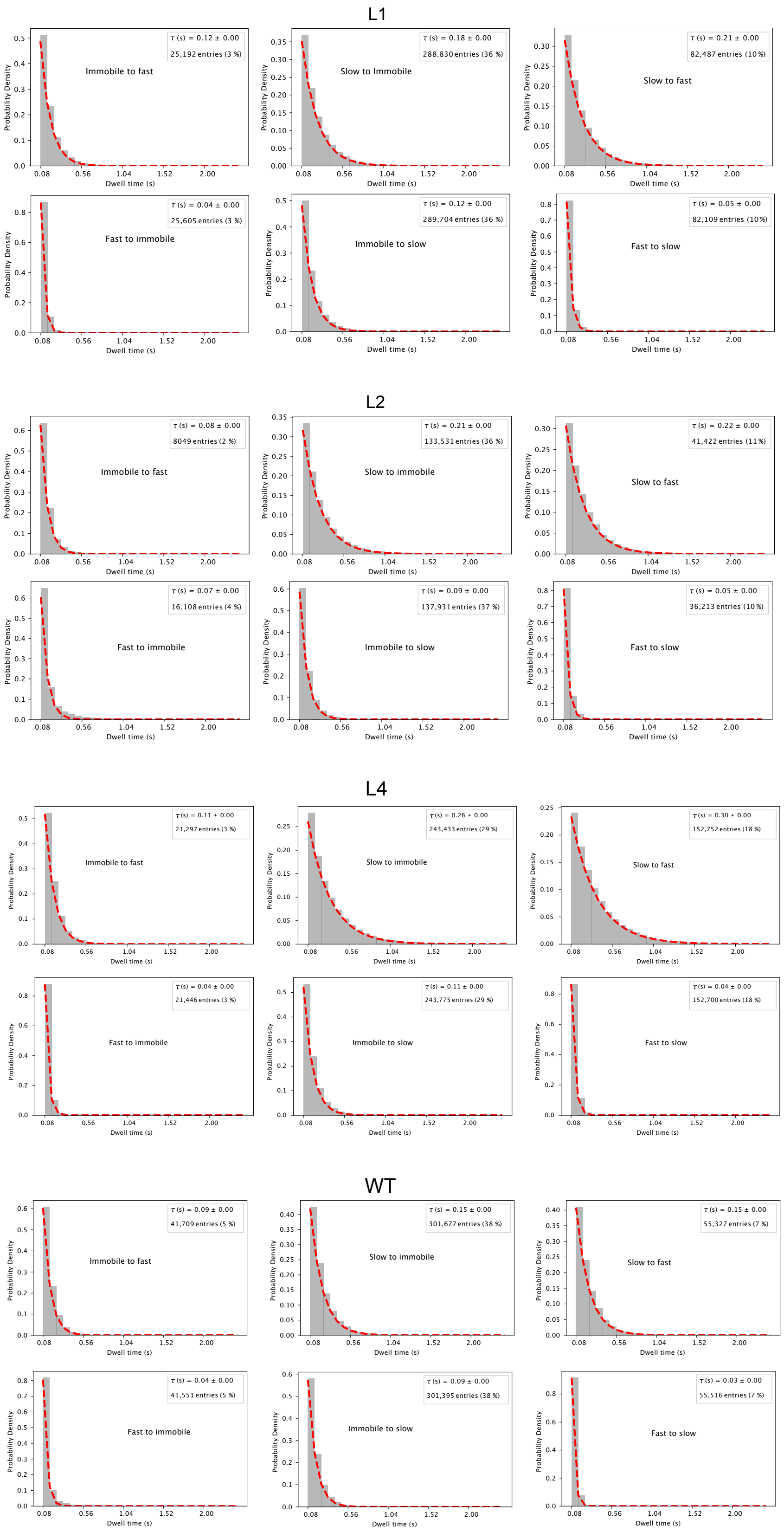

Figure S5. All transition lifetimes of TLL variants in the application condition, derived from TDPs using clustering. State dwell time histograms (gray) for all allowed transitions were fitted by an exponential decay (red) to report transition lifetimes. The bin size in the histograms is 81 ms (1 frame) per bin.

Table S2. Kinetic characterization of TLL variants in the application condition. Errors correspond to one standard deviation.

Lipase	Transition rates [s^{-1}]					
	**Immobile -> slow**	**Slow -> immobile**	**Slow -> fast**	**Fast -> slow**	**Fast -> immobile**	**Immobile -> fast**
**L1**	0.67 ± 0.002	0.44 ± 0.001	0.38 ± 0.002	1.73 ± 0.009	2.02 ± 0.017	0.68 ± 0.006
**L2**	0.91 ± 0.004	0.39 ± 0.002	0.37 ± 0.002	1.67 ± 0.012	1.09 ± 0.015	1.00 ± 0.014
**L4**	0.75 ± 0.002	0.31 ± 0.001	0.27 ± 0.001	2.01 ± 0.007	2.12 ± 0.019	0.74 ± 0.006
**WT**	0.86 ± 0.002	0.55 ± 0.001	0.52 ± 0.003	2.48 ± 0.015	1.87 ± 0.014	0.93 ± 0.006

Table S3. Transition energies of TLL variants. Errors correspond to one standard deviation.

Lipase	Transition energies, E_A [kJ/mol]					
	**Immobile -> slow**	**Slow -> immobile**	**Slow -> fast**	**Fast -> slow**	**Fast -> immobile**	**Immobile -> fast**
**L1**	73.973 ± 0.004	75.015 ± 0.004	75.378 ± 0.008	71.623 ± 0.007	71.239 ± 0.013	73.936 ± 0.014
**L2**	73.215± 0.000	75.314 ± 0.007	75.444 ± 0.012	71.710 ± 0.000	72.767 ± 0.000	72.981 ± 0.001
**L4**	73.694 ± 0.005	75.883 ± 0.005	76.225 ± 0.006	71.251 ± 0.005	71.119 ± 0.014	73.727 ± 0.016
**WT**	73.354 ± 0.004	74.462 ± 0.004	74.601 ± 0.010	70.731 ± 0.009	71.430 ± 0.011	73.161 ± 0.011
